# Improving Social Inclusion for People with Physical Disabilities: The Roles of Mobile Social Networking Applications (MSNA) by Disability Support Organizations in China

**DOI:** 10.3390/ijerph17072333

**Published:** 2020-03-30

**Authors:** Hyeon-Cheol Kim, Zong-Yi Zhu

**Affiliations:** 1 School of Business Administration, College of Business and Economics, Chung-Ang University, Seoul 06974, Korea; 2 Department of Arts and Cultural Management, Graduate School, Chung-Ang University, Seoul 06974, Korea; vampirenylon@gmail.com

**Keywords:** enjoyment experience, social network, online friendships, perceived social connectedness, well-being

## Abstract

Having friendships and interpersonal social connections is a normal and expected necessity of life that enhances an individual’s well-being. Digital platforms, such as mobile social network applications (MSNA), present a means for people with disabilities to integrate in society. This study combines intrinsic benefits (such as enjoyment experience and information) and extrinsic benefits (such as social networking) to explore the impact of these benefits on online friendships, and the influence of these online friendships on the perceived social connectedness and well-being of people with physical disabilities. The partial least squares methodology was used to conduct statistical analysis on survey data; the outputs were obtained through convergent analysis, discriminant analysis, and bootstrapping. The results showed that enjoyment experience and social network benefits significantly influence online friendships, which influence perceived social connectedness and well-being. In addition, the perceived social exclusion has a significant moderation effect. Our findings are expected to help local disability service organizations develop effective digital disability inclusion strategies to combat widespread social exclusion among people with disabilities.

## 1. Introduction

Having friendships and interpersonal social connections not only enhances an individual’s well-being but is also a normal and expected necessity of life. However, a large number of people with disabilities are socially excluded and are now out of the mainstream society. The issue of improving the well-being of people with disabilities has sustained attention from society and social welfare departments.

Digital platforms, such as mobile social network applications (MSNA), present a means for people with disabilities to integrate into society, from which they can obtain information benefits and enjoyment benefits without movement [1]. Furthermore, they can receive social support from online social interactions with others [1]. The benefits derived from social interactions reduce the sense of social exclusion of people with disabilities and offer many positive effects on overall psychological well-being. Considering the advantage of MSNA, the China Disable Persons’ Federation operates mobile social network applications that offer these benefits for people with disabilities to help improve their physical well-being. As a previous study [2] argued, well-being could moderate the negative effects of a physical disability and enhance a sense of belonging, which would promote mental health. 

Research on improving the well-being of people with disabilities through MSNA is under study. Most of the studies emphasized physically disabled persons’ MSNA usage statuses [3,4]. Such studies only investigated the descriptive statuses of MSNA usage through small-segment, face-to-face interviews or focus group interviews. However, not many studies applied statistical analysis to determine the relationships around MSNA usage. More recent studies are considering the relationship between physically disabled persons’ value/benefit needs, such as utilitarian or hedonic values, and MSNA usage [5,6,7], but they only examine intrinsic benefits. 

The most important function of MSNA is developing personal social networks that could help people build friendships with each other to enhance their well-being. However, research about the influence of MSNA’s extrinsic social network benefits on friendship building and well-being is still lacking. The usage studies on the disabled population are mainly focused on research garnered from reviewing documents or conducting face-to-face interviews. Statistical research about the disabled population’s mobile application usage is also limited and lacking.

To fill this important gap, this study combines intrinsic and extrinsic benefits to explore the impact of the benefits of MSNA on online friendships and the influence of online friendship on perceived social connectedness and well-being. In this study, benefits included enjoyment experience, information, and social network. Furthermore, this study added a moderator, i.e., perceived social exclusion, to explore its moderating effect and to determine whether MSNA helped reduce perceived social exclusion for enhancing the well-being of disabled people.

The goal of this study was to demonstrate how the benefits offered by MSNA help physically disabled persons develop online friendships, which could help their well-being. To achieve this goal, this study developed an integrated model to investigate clear relationships. While previous studies have drawn on informational benefits and hedonic benefits, this study used social network benefits as the antecedent of online friendships. This research also verified the need for these benefits given the level of social exclusion people with physical disabilities perceived. The results of this research will be offered for MSNA management to help people with physical disabilities improve their well-being and mental health. Our findings are expected to help local disability service organizations and the government to develop an effective digital disability inclusion strategy by responding to the widespread social exclusion among people with physical disability.

The rest of this paper is organized as follows. The literature review and hypotheses development are provided in Section 2. The study design is presented in Section 3. The empirical results are presented and discussed in Section 4. Finally, Section 5 is conclusions and Section 6 presents implications and outlines some limitations that provide opportunities for further study.

## 2. Research Hypotheses

### 2.1. Online Benefits and Online Friendship

The Internet offers various values for its users. In particular, it is an essential channel for people with physical disabilities to add and gain value. Perceived value and benefits refer to all assessments made by users for their service and can help to develop relationships and friendships with others [8]. Basically, perceived value involves the benefits that they can obtain from their activity. As mentioned above, MSNA is an important channel for people with physical disabilities to use to improve social interactions. In order to develop friendships or relationships with them, it is essential to offer various benefits. 

According to the existing literature [8,9], the perceived benefits could be divided into two dimensions, utilitarian benefits and hedonic benefits. Utilitarian benefits are also known as information benefits, which represent acquiring useful information from professional information providers or their friends. If the offered information is trustworthy, it will encourage users to develop relationships or friendships with the information provider. Users can then use the obtained information to solve any problems they face. Hedonic benefits are also known as enjoyment benefits, which refer to the pleasure, excitement, and enjoyment experience gained from interacting with others in the online environment or participating in entertaining activities. The joy element could help build relationships with others. Alternatively, information benefits and enjoyment experience benefits are of intrinsic value. The extrinsic value also exists. Extrinsic value includes social value and economic value [10]. Social value is assigned by individuals to their group membership [11]. For people with physical disabilities, the online environment is not only used to gain intrinsic value but is also used for building social networks. Following the social exchange theory, individuals engage in social interactions based on the expectation that they will lead, in some way, to social rewards, such as approval, status, and respect [12]. According to this theory, Wasko and Faraj [7] proved that one potential way an individual can benefit from active participation is based on the perception that participation can enhance their personal relationships and help them build friendships with others. 

The positive relationship between benefits and online friendships has been predicted in previous studies. Chen [13] attempted to engage digital media strategies for the purpose of studying interactive marketing and relationship management. The results showed that information value, entertainment value, and social network value positively influence relationship development, which could enhance online friendships with others. Further, Yoo et al. [14] conducted a study on 204 adult Twitter users with the intent of explaining how social value, utilitarian value, and hedonic value influence relationship development. The results indicated that social value positively influences relationships and friendship development. Similarly, Zhang et al. [8] offered insight to social networking site (SNS) managers on how the online relationship with users could be enhanced to continue their relationship. The results showed that social value and enjoyment value significantly influenced a continued relationship, but the information value did not. Van Tonder et al. [15] focused on the influence information value had on relationships. This research collected 511 electronic banking consumers and explored how information benefits can be positively associated with relationships. Itani et al. [16] drew on the hedonic benefits value and showed a positive relationship. Online researchers [17,18] also interviewed people with physical disabilities, showing that the online environment helped them build relationships or friendships with others. Thus, this study hypothesizes that online benefits can positively affect online friendships, and these hypotheses are detailed as follows:

**Hypothesis 1** **(H1).** *Online enjoyment experience benefits can positively affect online friendship*.

**Hypothesis 2** **(H2).** *Online information benefits can positively affect online friendship*.

**Hypothesis 3** **(H3).** *Online social network benefits can positively affect online friendship*.

### 2.2. Online Friendship and Perceived Social Connectedness

In the offline interpersonal communication study, a full friendship development process can be typically defined as one in which a stranger evolves into an acquaintance, into a new friend, and then into a close friend [19,20]. For people with physical disabilities, friendships develop in the same way. Trust plays an essential role in the friendship development process [21]. If there is no trust, the friendship will not be developed. Thus, online friendships are the same as offline friendships. According to Sheer [20], online friendships involve a process in which an individual encounters new people, obtains information, experiences enjoyment about people of interest, gets to know them, and develops and maintains relationships. 

In the previous literature, online friendships were regarded as an important factor affecting individuals’ sense of belonging. Perceived social connectedness is defined as the quality of connections an individual has with other people in their social circle, that is, the sense of them belonging to one community [22]. Social connectedness is a reflection of an internal sense of belonging and closeness with the individual’s social world, including relationships with friends, family, peers, acquaintances, strangers, the community, and society [23]. Grieve et al. [24] investigated whether online friendship can help develop perceived social connectedness. This research indicated that the use of Facebook can provide opportunities to develop and maintain friendships, and, thus, maintain social connectedness. Moreover, other research used international students to demonstrate the influence of online friendships on their perceived social connectedness and life satisfaction. This result indicated that the ratios, strength, and variability of online friendships influence their perceived social connectedness and decrease their homesickness [25]. The online benefits for people with physical disabilities, and the influence of online communication on their life, were also investigated. Shpigelman and Gill [26] determined that online communication can help build online friendships and could help develop perceived social connectedness. Thus, this study hypothesizes the following:

**Hypothesis 4** **(H4).** *Online friendship can positively affect perceived social connectedness*.

### 2.3. Online Friendship and Well-Being

Well-being refers to the quality of an individual’s life, which includes happiness and life satisfaction [27]. As Ellison, Steinfield, and Lampe [28] argued, the reason MSNA is popular in many countries is that it enables users to efficiently extend and maintain friendship networks. The central functions of MSNA are to connect friends, develop online friendships, and obtain information needs and joy. As MSNA communication does not require physical movement, it is an efficient way for people with physical disabilities to communicate with others, and, thus, helps them enhance their well-being. Other researchers [29,30,31] have revealed that online communication behavior was associated with well-being. 

Grigorescu [32] studied young consumers’ online communications, and the results indicated that online friendships can form a powerful barrier against stressors, thereby helping young consumers develop well-being. Similarly, Goswami [33] focused on children and determined the influence of negative online friendships and positive online friendships on children’s well-being. This study also indicated that positive online friendships increased their well-being, whereas negative online friendships decreased their well-being. Additionally, there was evidence for the relationship between SNS users and well-being. The results found that online friendships can reduce user stress and can help users develop their own well-being [6]; this well-being could help disabled persons be in better health [2]. Online channels are an important and essential channel for people with physical disabilities due to little physical movement being required. Online channels could help improve their quality of life. Previous studies also argued that online-based communication gives them the chance to communicate with others, and online friendships were positively associated with their well-being [34]. Thus, this study hypothesizes the following:

**Hypothesis 5** **(H5):** *Online friendship can positively affect well-being*.

### 2.4. Perceived Social Connectedness and Well-Being

Perceived social connectedness is the perceived sense of belonging or connectedness. This perceived connectedness is a basic psychological need, according to self-determination theory, and, when satisfied, brings about positive outcomes, such as well-being [35]. Baumeister and Leary [36] argued that connectedness is a fundamental motivation, functions in a wide variety of settings, and is essential for well-being. Deci and Ryan [37] revealed that satisfying this need could foster well-being. Therefore, we assume that perceived social connectedness is an essential factor for well-being. 

Previous studies have predicted the positive relationship between perceived social connectedness and well-being. Yoon et al. [14] proved that social connectedness positively affected well-being for Asian Americans. Paul, Ryan, and Pryor [35] investigated whether or not social connectedness predicts well-being in adolescence. The results also determined that social connectedness affected well-being. In the online environment, Morris et al. [38] drew from their perspective on smart technologies, proving that smart technologies can help enhance social connectedness in the elderly. Improving or maintaining social connectedness can enhance their well-being and reduce their isolation and loneliness. The research of Obst and Stafurik [34] on disabled persons also showed that a sense of belonging in a community was positively associated with well-being. Thus, this study hypothesizes the following:

**Hypothesis 6** **(H6).** *Perceived social connectedness can positively affect well-being*.

### 2.5. Perceived Social Exclusion Moderation Effect

Reducing perceived social exclusion is an important goal of social life and economic policy. Social exclusion is defined as a process whereby certain individuals are pushed to the edge of society and prevented from participating fully by virtue of their poverty, by lack of basic competencies and lifelong learning opportunity, or as a result of discrimination [39]. Social exclusion happens when different factors combine to trap individuals and areas in a spiral of disadvantage [36]. People with physical disabilities have disadvantage in movement. The impaired movement may impact their feeling of exclusion in the main society. As previous argued, social exclusion affects people’s well-being [40]. Perceived social exclusion may enhance disabled online behavior without any movement. Thus, this study hypothesizes the following:

**Hypothesis 7** **(H7).** *Perceived social exclusion has a moderation effect in the path*.

**Figure 1:**
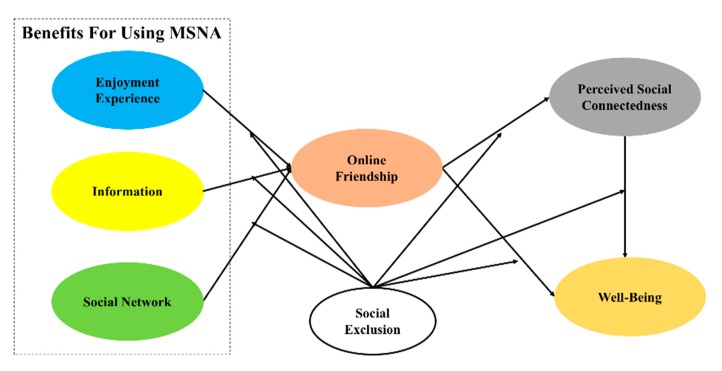
Research model.

Our proposed research model, based on previous studies, is presented in Figure 1. This conceptual model includes the benefits from using MSNA (enjoyment experience, information, social network); online friendship; perceived social connectedness; and well-being. Thus, a total of seven research hypotheses related to the relationships among these variables were formulated (H1–H7).

## 3. Material and Methods

### 3.1. Study Design and Participants

**Figure 2:**
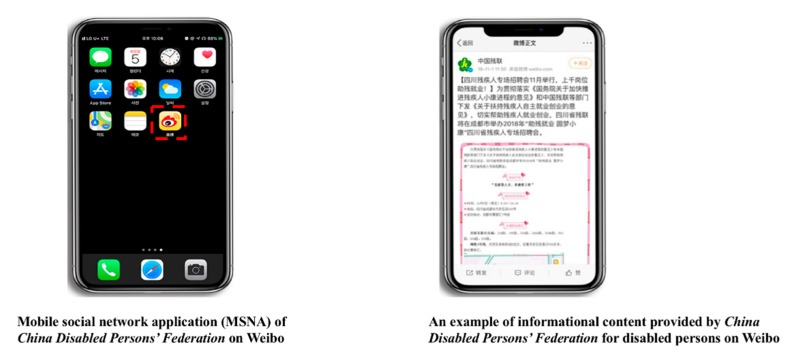
Mobile social network application by a disability service organization.

This study used an integrated conceptual design as a model construction study for verifying the research model. The participants of this study were people with physical disability who have been active users of the China Disabled Person’s Federation’s mobile social network application (MSNA) in Chengdu, Sichuan province, People’s Republic of China for more than six months; see Figure 2. In this research, we only considered people with certain physical disabilities, that is, only those with movement and mobility impairments and hearing impairment. Mental health disability and vision impairment were not included. Moreover, those with cognitive impairments, which may have hindered adequate understanding of the survey questions, were excluded from this study. Inclusion criteria were those who could understand the purpose of this study, had the cognitive ability to respond, and signed the informed consent form for study participation to signify their voluntary consent to participate in the study. First, the researcher conducted personal interviews with each participant to check whether the participant is a user of the official disabilities on MSNA in the past six months and determine their normal MSNA behavior. What they think about the MSNA was also investigated. After personal in-depth interviews, more specific questions were asked in the questionnaire survey. The participants were asked to follow a unique web link to the survey questionnaire and complete the survey. Considering the human rights of disabled persons and the physical difficulties they face, the survey did not include any questions that may violate their rights. The survey completion time was restricted to one hour. Finally, in this study, a total of 104 valid survey respondents were chosen for the analysis, of which 43.3% were male and 56.7% were female. All participants were experienced followers of MSNA.

### 3.2. Measures

A self-administered questionnaire was used to collect the data. It included questions related to the enjoyment experience benefit, information benefit, social network benefit, online friendship, perceived social connection, well-being, social exclusion, and demographic information in the context of MSNA. The questionnaire was developed in three steps. First, all the constructs were measured by applying multiple-item perceptual scales with pre-validated instruments based on previous studies. Enjoyment experience benefit included four items from Koufaris [41] and Zhou, Li, and Liu [42]. Information benefit consisted of three items adapted from Ducoffe [43] and Logan et al. [44]. Social network benefit included four items based on Arnold and Reynold [45] and Yen [46]. Online friendship was measured by three items adapted from Roberts, Varki, and Brodie [47] and Zhou et al. [8]. Perceived social connection was measured by four items from Chen [13] and Han, Min, and Lee [48]. Well-being was measured by three items developed by Pavot and Diener [49]; Samaha and Hawi [50]; and Kong, Ding, and Zhao [51]. Social exclusion was measured with four items from Huxley et al. [52] and Lim and Kim [53]. 

Second, the questionnaire was first developed in English and then translated into Chinese by a researcher who is fluent in both English and Chinese. The Chinese version of the survey was then translated back into English by a native Chinese professor who is also fluent in both English and Chinese to ensure translation equivalence. 

Third, to verify the content’s validity and fitness in the context of persons with disabilities, the researcher conducted focus group interviews with two experts from the China Disabled Persons’ Federation as well as one teacher with a physical disability; they were asked to evaluate whether the items were appropriate to assess persons with disabilities. Moreover, the experts were asked to evaluate the survey items to determine whether the measurement items needed to be deleted or reworded and to suggest items that should be added, if necessary. 

All items were measured on a five-point Likert scale that ranged from strongly disagree (1) to strongly agree (5).

### 3.3. Procedures

A preliminary survey was conducted from April 2019 to May 2019 to determine the questionnaire measurement tools that would be used in this study. A preliminary survey was administered to 30 MSNA users who were physically disabled and identified as having used MSNA in the past six months. Exploratory factor analysis, confirmatory factor analysis, and a reliability test for preliminary items, including demographic characteristics and cognitive ability items, were conducted. The results of the preliminary tests showed that the questionnaire results had meaning, relevance, and clarity. The main survey was conducted from June 2019 to July 2019.

### 3.4. Statistical Analysis

This research selected SmartPLS, a software that makes models using partial least squares (PLS), to produce the statistical analysis that would verify the research model [54] “since the PLS algorithm is a components-based structural equation modeling technique, which allows the indicator to vary in how much it contributes to the composite score of the latent variable”. In addition, PLS is an essential form of exploratory analysis for finding new relationships in research. The SmartPLS results are obtained through the following three steps: (1) convergent analysis; (2) discriminant analysis; and then (3) bootstrapping to examine the hypotheses.

## 4. Results

### 4.1. Measurement Model

First, using confirmatory factor analysis (CFA), the measurement model was tested by content validity, convergent validity, and discriminant validity. This study adapted the measurement items and the constructs from the prior studies and conducted the content validity from pre-tests. In addition, we examined factor loading, Cronbach’s alpha, composite reliability (CR), and average variance extracted (AVE) to evaluate the convergent validity. Table 1 and Table 2 show the factor loading, CR, and AVE. Each item of factor loading is higher than the acceptable threshold of 0.7, the Cronbach’s alpha and CR of each construct is higher than the acceptable threshold of 0.7, and the AVE for each construct is higher than the acceptable threshold of 0.5. Thus, convergent validity is supported. This study tested inter-construct correlation coefficients to measure the discriminant validity. The square roots of the AVEs for each construct in bold are higher than the values in the corresponding columns and rows, verifying the discriminant validity. Therefore, on the basis of the above outcomes, the measurement model is validated. 

### 4.2. Structural Model and Discussion

**Figure 3:**
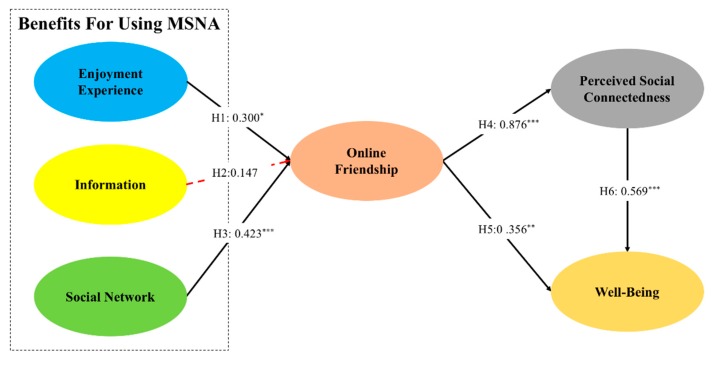
Results of hypothesis (*N* = 104). * *p* < 0.05; ** *p* < 0.01; *** *p* < 0.001.

As shown in Table 3, most of the hypotheses are validated by the data samples. Specifically, enjoyment experience benefit (β = 0.300, *p* < 0.05) and social network benefit (β = 0.427, *p* < 0.000) positively influence online friendship, which supports H1 and H3, respectively. Online friendship is found to positively influence the perceived social connectedness (β = 0.876, *p* < 0.000) as well as well-being (β = 0.356, *p* < 0.000), which supports H4 and H5, respectively. Moreover, perceived social connectedness positively influences well-being (β = 0.569, *p* < 0.000), which means H6 is supported. However, information benefit does not significantly influence online friendship (β = 0.147, *p* > 0.05), so H2 is not supported. Figure 3 presented the results of hypothesis testing.

**Figure 4:**
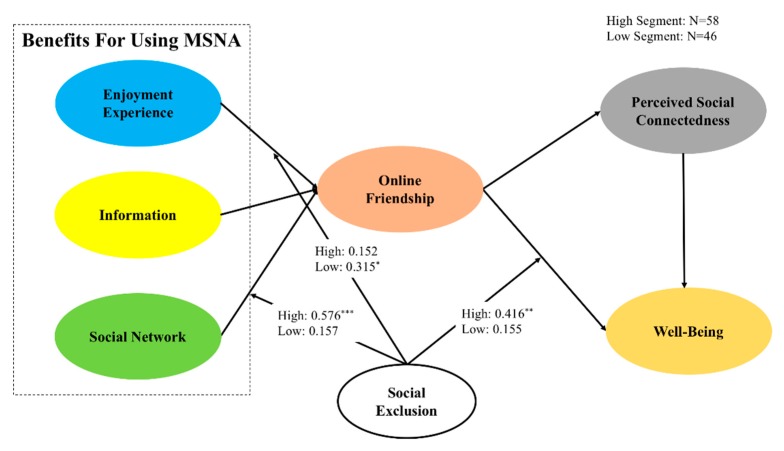
Results of mediation effect. * *p* < 0.05; ** *p* < 0.01; *** *p* < 0.001.

With regard to the moderator in this model, the results also confirm the moderation effect. We examined the total score of perceived social exclusion measures and evaluated the average score of each total score. We then divided the participants into two segments: high perceived social exclusion (*N* = 58) and low perceived social exclusion (*N* = 46). Perceived social exclusion moderates the relationship between the benefits and online friendship and between online friendship and well-being, as shown in Figure 4.

## 5. Conclusions

This study intended to demonstrate how the MSNA helps in maintaining online friendships of physically disabled persons, which could improve their well-being. This study explored the benefits of MSNA regarding online friendship, perceived social connectedness, and well-being of people with physical disability; MSNA was found to be helpful in letting them live healthier lives. Following previous studies, a research model with seven hypotheses was developed in this study to determine the abovementioned relationship. To test these hypotheses, this study conducted a survey with 104 valid respondents who were all users of MSNA. SmartPLS was chosen for the statistical analysis. Six of the hypotheses were validated and thus confirmed this significant relationship. To be specific, the results of this study indicated that enjoyment experience and social network benefits have significant positive influences on online friendship, while the influence of information benefit is not significant. Thus, H1 and H3 were supported, and H2 was not supported. This study also showed that online friendship significantly influences users’ perceived social connectedness and well-being. Moreover, perceived social connectedness significantly influenced well-being. Based on these results, H4, H5, and H6 were supported. The results of the moderator, perceived social exclusion, showed that the significant moderation effect was accurately predicted. Thus, H7 was supported. The theoretical and managerial implications will be discussed later.

## 6. Implications

### 6.1. Theoretical Implication

First, the previous studies related to friendship development drew on the intrinsic factors (utilitarian and hedonic benefits). This study predicted the intrinsic information, enjoyment benefit, and extended the original model by adding whether the social value influences online friendship and relationship development. The online environment is used as an intrinsic motivation by people with physical disabilities. Extrinsic motivation is also an essential factor. The results indicated that enjoyment benefit and social benefit were significantly associated with online friendship development. Previous research also determined that enjoyment benefit is positively related to the online friendship development [16]. Social network benefit was also predicted to enhance the online friendship development [13,55]. However, the information benefit did not significantly influence online friendship development. Some previous research argued that information benefit is the elementary factor for influencing relationship development [15,16]. This research explored the different results. This research drew on physically disabled persons’ experiences, while most of the existing studies focused on persons without disabilities. The different research participants produced different outputs. Further, Zhang et al. [8] determined that information benefit can influence online friendship, but not significantly. Thus, this result is also meaningful. 

Second, this study explored the influence of online friendship on perceived social connectedness and well-being. The results showed that online friendship positively influences perceived social connectedness and well-being, and online friendship influences well-being through perceived social connectedness. These results are the same as the previous studies, as the previous studies argued that online friendship can help users enhance their sense of belonging in a community [20,24,25]. This study confirmed this proposition. The result of online friendships helping to develop well-being was also confirmed in previous studies [6,32,34]. The result of perceived social connectedness impacting well-being was also found in previous studies [34,35,38]. 

Third, this study added the moderator, perceived social exclusion. People with physical disabilities may find it hard to move. Most of them show high levels of social exclusion, which can lead to a relatively low life quality. An online environment offers them space, which could reduce their perceived social exclusion. The results show that a high level of social exclusion has a significant effect on online friendships; on the other hand, users with a low level of social exclusion prefer information benefit. 

### 6.2. Managerial Implication

From a practical standpoint, community disability services practitioners who seek to motivate individuals to interact with people with physical disabilities need to frame their mobile communication channels in a way that draws systematic support for the well-being of disabled persons and their achievement of social inclusion through information access. The results showed that the online environment is an essential platform for persons with disabilities to communicate. People with physical disabilities can obtain enjoyment experience, information, and social networked benefits. To enhance the lives of people with physical disabilities, community disability services practitioners should offer them more enjoyment and social network benefits. Such offers can provide interesting features and social network development opportunities that would improve their quality of life. Considering these benefits, people with physical disability can easily continue their use in their daily life. 

More importantly, the online mobile communication channel can help people with physical disabilities enhance or maintain their social relationships with others to ensure their well-being. These findings reconfirmed the necessity to utilize the mobile communication channel for people with physical disability, as these people are found to be reliant on chatting software on mobile smart devices as a necessity. Online Chinese foundations for people with disabilities should expand their services to increase the latter’s social connectedness, which is an important factor for their well-being. Next, it is necessary to increase the promotion of the Chinese online communication channel, which could enhance the life satisfaction of persons with disabilities. If they have the opportunity to communicate with others, they could maintain friendships without any movement. Thus, since the online channel can be useful for people with physical disabilities who are interested in online information or social interaction, the roles of the government and online Chinese disability foundations are crucial. An online social network will help people with physical disabilities improve their well-being. In addition, they are able to conduct more types of activities in the MSNA environment, such as disability-related online business and online education. Thus, creation of MSNA accounts for people with disabilities is necessary. 

### 6.3. Limitation

Based on a survey, this study found that MSNA benefits and improves the well-being of Chinese people with disabilities involving movement and mobility impairments. Our findings showed that MSNA can benefit people with physical disability build or maintain relationships to improve their social connectedness and well-being. However, this study has certain limitations. To address them, we recommended that the following should be considered in future research on the MSNA usage behavior of people with disabilities. First, this study’s findings cannot be generalized to a large population of people with physical disability, because it utilized data from only Chengdu, Sichuan. Moreover, this study’s participants did not associate with demographic factors, such as gender and education. Second, more representative variables need to be considered for people with disability. It is necessary to understand that there exist various types of disabilities beyond those considered in this research, such as mental health disability and vision impairment. In addition, this study considered only the most important factors related to disabilities, but future studies should consider other factors, such as whether the population with disabilities is able to receive social support in an online environment. Cho and Lee [1] had argued that the population with disabilities needs online social support to develop a sense of belonging. Further, online environmental factors should be examined to design a more comfortable usage environment for persons with disabilities. Third, this study examined the moderating effect of perceived social exclusion. Examining the intensity of other moderators of SNS usage and off-line relationship quality, especially age, gender, level of physical disability, and social exclusion, could also be meaningful.

## Figures and Tables

**Table 1:** Measurement items and factor loading.

Construct	Measurement	Factor Loading	Source
Enjoyment Experience Benefit (EEB)	MSNA is fun.	0.882	Koufaris [41] and Zhou, Li, and Liu [42]
	MSNA is exciting.	0.883	
	MSNA is enjoyable.	0.901	
	MSNA is interesting.	0.921	
Information Benefit (IB)	MSNA is a good source of information.	0.877	Ducoffe [43] and Logan et al. [44]
	MSNA supplies relevant information.	0.897	
	MSNA provides timely information.	0.910	
Social Network Benefit (SNB)	I can contact friends on MSNA.	0.839	Arnold and Reynold [45] and Yen [46]
	I can share experiences with friends on MSNA.	0.891	
	I can develop friendships with others on MSNA.	0.821	
	I can extend personal relationships on MSNA	0.840	
Online Friendship (OF)	The MSNA provider is trustworthy.	0.895	Roberts, Varki, and Brodie [47] and Zhou et al. [8]
	I am happy with the MSNA provider’s performance	0.908	
	I continue to deal with the MSNA provider because I genuinely enjoy my relationship with them.	0.912	
Perceived Social Connectedness (PSC)	I feel I am connected to other users on MSNA.	0.847	Chen [13] and Han, Min, and Lee [48]
	I feel like I fit in on MSNA.	0.888	
	I have made connections with other people on MSNA.	0.896	
	I feel comfortable communicating with other people on MSNA.	0.805	
Well-being (WB)	The conditions of my life are excellent.	0.880	Pavot and Diener [49], Hawi [50], and Kong, Ding, and Zhao [51]
	I am satisfied with my life.	0.865	
	So far, I have achieved the important things I want in life.	0.901	

**Table 2:** Reliability and inter-construct correlations.

Construct	Cronbach’s Alpha	CR	AVE	WB	EEB	IB	OF	PSC	SNB
WB	0.858	0.913	0.778	0.882					
EEB	0.905	0.934	0.779	0.775	0.882				
IB	0.946	0.957	0.786	0.778	0.890	0.887			
OF	0.889	0.931	0.819	0.855	0.761	0.751	0.905		
PSC	0.882	0.919	0.740	0.882	0.774	0.771	0.878	0.860	
SNB	0.870	0.911	0.719	0.788	0.776	0.778	0.773	0.761	0.848

CR: composite reliability; AVE: average variance extracted.

**Table 3:** Results of hypothesis pathway.

Hypothesis	Estimate (Standard)	CR	*p*-Value	Support
H1	0.300	2.310	0.02	Yes
H2	0.147	1.108	0.268	No
H3	0.423	4.380	***	Yes
H4	0.876	18.427	***	Yes
H5	0.356	3.958	***	Yes
H6	0.569	6.319	***	Yes
